# Development of language and arithmetic skills: risk and protective factors. Comparative cross-sectional study

**DOI:** 10.1590/1516-3180.2020.0280.r1.10122020

**Published:** 2021-03-12

**Authors:** Patrícia Aparecida Zuanetti, Marina Dias Macedo de Melo Avezum, Marita Iannazzo Ferretti, Angela Cristina Pontes-Fernandes, Marina Estima Neiva Nunes, Nelson Macedo Liporaci, Marisa Tomoe Hebihara Fukuda, Ana Paula Andrade Hamad

**Affiliations:** I PhD. Speech Therapist, Department of Health Sciences, Faculty of Medicine of Ribeirão Preto, Universidade de São Paulo, Ribeirão Preto (SP), Brazil.; II BSc. Psychologist, Department of Neuroscience and Behavioral Sciences, Faculty of Medicine of Ribeirão Preto, Universidade de São Paulo, Ribeirão Preto (SP), Brazil.; III BSc. Psychologist, Department of Ophthalmology, Otorhinolaryngology and Head and Neck Surgery, Faculty of Medicine of Ribeirão Preto, Universidade de São Paulo, Ribeirão Preto (SP), Brazil.; IV PhD. Psychologist, Department of Neuroscience and Behavioral Sciences, Faculty of Medicine of Ribeirão Preto, Universidade de São Paulo, Ribeirão Preto (SP), Brazil.; V MD. Department of Neuroscience and Behavioral Sciences, Faculty of Medicine of Ribeirão Preto, Universidade de São Paulo, Ribeirão Preto (SP), Brazil.; VI MD. Department of Neuroscience and Behavioral Sciences, Faculty of Medicine of Ribeirão Preto, Universidade de São Paulo, Ribeirão Preto (SP), Brazil.; VII PhD. Speech Therapist, Associate Professor, Department of Health Sciences, Faculty of Medicine of Ribeirão Preto, Universidade de São Paulo, Ribeirão Preto (SP), Brazil.; VIII MD, PhD. Assistant Professor, Department of Neuroscience and Behavioral Sciences, Faculty of Medicine of Ribeirão Preto, Universidade de São Paulo, Ribeirão Preto (SP), Brazil.

**Keywords:** Language development disorders, Risk factors, Academic performance, Social skills, Problem behavior, Environment resources, Arithmetic, Prenatal, perinatal, and postnatal complications

## Abstract

**BACKGROUND:**

In a literate society, linguistic/arithmetic performance is highly valued. Based on defined risk factors, strategies for promotion of better performance can be developed.

**OBJECTIVE:**

To ascertain the risk and protective factors relating to development of language and arithmetic.

**DESIGN AND SETTING:**

Observational comparative cross-sectional study at a public elementary school in Ribeirão Preto (SP), Brazil.

**METHODS:**

A total of 66 children (41% females) attending first to fifth grades participated in this study. They were divided into two groups: G1, children classified as presenting language or arithmetic deficits; G2, average performance. Language (oral and written) and arithmetic skills were assessed through standardized tests. Variables relating to social skills, home environment resources and behavioral problems were assessed through standardized scales. Data on other variables (pre, peri and postnatal complications, maternal variables and others) were collected through interviews. The logistic regression technique with LASSO was used (α = 0.05).

**RESULTS:**

Teenage pregnancy and consumption of psychoactive substances during pregnancy or complications during pregnancy were risk factors for performance regarding arithmetic and language. Higher schooling level for the mother was a protective factor in the development of arithmetic and language. Being female and having a history of otitis were risk factors for language. Altered social skills (responsibility and civility) and complaints of inattention were risk factor for arithmetic. Adequate linguistic development was a protective factor for the development of arithmetic.

**CONCLUSION:**

The risk/protective factors included variables relating to the gestational period, mother's age when pregnant, mother's schooling, social skills, behavior and development issues.

## INTRODUCTION

Language is a complex cognitive function, characterized by a system of principles and rules that enable people to code their meaning in a symbol, and vice-versa.^1^ It has both expressive and receptive components.^1^ Under a natural course, oral language skills are developed first (pragmatic, phonological, morphosyntactic and semantic aspects), and then metalinguistic and written language skills are acquired.

Arithmetic skills, in turn, are related to numerical properties and operations.^1^ To develop arithmetic skills, three different systems are recruited, depending on the task: a nonverbal system (responsible for presenting the relationship between numbers); a verbal system (the numbers are expressed as a type of word); and a visual system (numbers can be decoded as Arabic numerals).^2^

Development of the abovementioned cognitive skills is of utmost importance to the life of every human being within literate society.^3^ For these skills to be adequately developed, children need to be integrated into various environments, and to acquire other levels of development.

Recent reports have shown prevalences of alterations in written language and/or arithmetic performance of approximately 22% among Indian schoolchildren^4^ and 54% among Brazilian schoolchildren.^5^ Regarding the prevalence of specific learning disorders (dyslexia and/or dyscalculia), the same studies stated that the percentage was 3% in Brazil,^5^ 9% in Turkish-born children^6^ and 7.5% in India^4^. They also showed that high rates of comorbidities were present in children with complaints of reading, writing and/or arithmetic difficulty: the conditions most present were attention-deficit/hyperactivity disorder and mood disorders.^5^^,^^6^

Despite variability from country to country regarding the prevalence rates of low school performance, researchers share the unanimous opinion that early intervention should be implemented among these children in order to promote adequate social and cognitive development.^5^^,^^6^ To this end, the risk and protective factors concerning language and arithmetic development need to be identified.

By definition, risk factors encompass negative events that take place in one's life; when these are present, the likelihood of having physical, social or emotional problems increase.^7^ Protective factors, on the other hand, are those that are capable of protecting people who are at risk (thereby reducing the impact of risks and the negative chain reactions that follow the person's exposure to a situation of risk); these can be both individual and environmental factors.^8^

The studies in the literature regarding risk factors for language and/or arithmetic skills have mostly analyzed one variable at a time, e.g. studies on prematurity,^9^^,^^10^ severe malnutrition in the first years of life^11^ or resources present at home,^12^^,^^13^ among others. However, few studies have analyzed which variables out of these many factors are the most likely to impair linguistic and mathematical development.

## OBJECTIVE

The aim of study was to assess several variables – maternal and environmental variables, pre, peri and postnatal conditions and social skills/behavioral problems – and ascertain which of these are the risk and protective factors for development of language and arithmetic skills.

## METHODS

This was an observational comparative cross-sectional study. It was approved by our institution's human research ethics committee (evaluation report: 3.774.559; certificate CAAE: 26465219.8.0000.5440; date: December 16, 2019).

### Sample characterization and selection

All the children studying in the first to fifth grades in one public elementary school were eligible to participate in this study. For this, prior authorization from the adults responsible for them was required. Firstly, the parents/guardians (approximately 170 in number) were invited to participate in a meeting at which the project was explained; then, they were sent a consent form to authorize the minors’ participation in this study. Out of the 170 invitations, 77 parents/guardians authorized their children to participate; however, based on the inclusion and exclusion criteria, the final sample comprised 66 children.

The inclusion criteria were that the children needed to be 6.5 years to 11 years old, attending that elementary school at first to fifth-grade level, and did not present any syndrome or pathological condition that impaired their cognitive capacity, e.g. fetal alcohol syndrome, Down syndrome or others. The exclusion criteria encompassed situations in which the parents/guardians did not fill out the questionnaires used in this study and the assessments on linguistic and arithmetic skills were incomplete.

Among the 66 children participating in this study, 27 (41%) were female. The children's mean age was 8.3 years (standard deviation 1.3). Regarding their school year, 8 (12%) were attending the first year; 12 (18%), the second year; 24 (36%), the third year; 13 (20%), the fourth year; and 9 (14%), the fifth year.

Based on these children's classification concerning linguistic and arithmetic skill performance for their age, they were divided into two groups, namely:

- Group 1 (G1): children classified as borderline or deficient (any degree) in the language test (oral and written) or arithmetic test (depending on the variable studied in the statistical model);
- Group 2 (G2): children whose performance was classified as average or superior for their age in the language test (oral and written) or arithmetic test (depending on the variable studied in the statistical model).

### Data collection instruments and procedures

The data collection was divided into two parallel phases. The first one involved oral and written language and arithmetic skills assessments, and used a standardized, validated instrument for the Brazilian population (see description below), individually administered at the school.

The second phase investigated occurrences of several variables that might interfere with linguistic and arithmetic development. These variables are described in **Table 1**. A brief interview was conducted with the parents/guardians to collect this information; they were then asked to fill out certain questionnaires. These instruments are described below.

**Table 1:** Variables selected for this study

Identificationdata	Date of birth Current age Gender
**Maternal and social variables**	Mother's age during pregnancy (< 18 years = teenage pregnancy) Consumption of psychoactive substances during pregnancy (alcoholic beverages, tobacco and other drugs) Complications during pregnancy (e.g. physical trauma, surgery, bleeding and others) Mother's schooling level Home environment resources^14^
**Birth and postnatal conditions**	Gestational age (preterm, full-term or post-term) Size for gestational age (weight and length) Birth complications Delay in neuropsychomotor development Delay in speech and language development Epilepsy and other conditions in early childhood (malnutrition, anemia, etc.) Current complaints of sleep alterations History of recurrent otitis or serous otitis in early childhood
**Social behavior/ skills**	Alterations in social skills^15^ Alterations in behavior (SSRS and SNAP)^15,16^

The instruments administered for data collection (variables listed in **Table 1**) are described in sequence, along with the linguistic and arithmetic performance assessment tests.

- Child Neupsilin test:^1^ This is a brief neuropsychological instrument that, through 26 subtests, assesses components of eight neuropsychological functions: orientation; attention; visual perception; working, episodic, and semantic memory; arithmetic skills; oral and written language; visuoconstructive abilities; and executive functions. It can be administered to children from 6 to 12 years old, and only by professional psychologists or speech-language-hearing therapists.

The items administered in this study were language (oral and written) and arithmetic skills, which are composed of the following subitems:

(a1) Oral language (pragmatic, lexical-semantic and phonological aspects, and metalinguistic skills): picture-naming task; phonological awareness, i.e. rhyming and phoneme deletion; and oral comprehension and inferential processing.
(a2) Written language: tasks involving reading syllables, words and pseudowords aloud; writing words and pseudowords; understanding written sentences; spontaneously writing a sentence; and copying a written sentence.
(b) Arithmetic skills: counting sticks and doing arithmetic calculations.

In each item, the child's performance is classified according to their age, by calculating the Z score (number of standard deviations above or below the population's mean value). This classification was used to form the groups. Children whose performance was classified as “borderline” (Z score between −1 and −1.49) or “deficient” (Z score below −1.5) were classified as having altered performance. On the other hand, those with a Z score between −0.99 and +1 were classified as average and thus were placed in group 2.

- Printed interview: A brief questionnaire structured by the researchers was directed to the parents/guardians in order to gather information relating to the child's medical history, gestational complications, cognitive, motor and linguistic development, history of recurrent otitis, hearing complaints, food-related complaints, school complaints, sleep alterations and other matters. Before each adult answered it, the researchers personally explained the questionnaire and clarified any questions that they might have.
- **Home Environment Resources Scale (HERS):**^14^ This instrument evaluates the resources at home that might contribute to academic learning during elementary school. It encompasses three domains:
  - resources that promote proximal processes;
  - activities that indicate stability in family life;
  - parental practices that help to connect family and school.

This instrument was administered as a semi-structured interview, in which each topic was presented orally to the interviewee. The interviewer was free to paraphrase the question if there was any difficulty in being understood.

It has ten questions: seven that are open-ended (with items for the parent/guardian to mark) and three on a three-point scale represented by 0 (never), 1 (sometimes) and 2 (always).

For this study, the crude score was used, i.e. each item marked in the first seven questions received one point. This score was added to that of the last three questions, which used the scale. The higher the score was, the more resources that there were in that home.

- **Social Skills Rating System (SSRS):**^15^ This is a validated precise instrument for mapping social skills and behavioral alterations, and for monitoring the effectiveness of interventions aimed at the socioemotional development of children and adolescents.

This instrument enables information from three different sources – the child, the parents and the teachers – to be collected and compared. However, for this study, only the questionnaire approaching the parents was used.

The parents’ questionnaire is composed of 38 questions: 23 relating to social skills (measured in terms of frequency, considering affectivity/cooperation, responsibility, self-control, civility and social interaction); and 15 relating to behavioral problems (assessed in terms of how frequently they occurred, considering both externalizing and internalizing behaviors).

The parent/guardian was asked to answer the questions on these two frequency-related scales as follows: 0 (never), 1 (sometimes) or 2 (very frequently). Afterwards, the score was calculated and compared with the percentile indicated for the test, to verify whether the child had a behavioral and/or social skill alteration. In this study, a social skill was considered altered when the child was classified below the 25^th^ percentile and behavior was considered altered when the child was classified over the 75^th^ percentile, as recommended in the instructions for this test.

- **SNAP-IV questionnaire:**^16^ This is an assessment instrument that is used to track and assess the frequency of inattention, hyperactivity and impulsivity symptoms, based on the criteria of the fourth edition of the Diagnostic and Statistical Manual of Mental Disorders (DSM-IV), of the American Psychiatric Association. The Portuguese-language version of SNAP-IV was validated for use in Brazil by Mattos et al.^16^ Even though this translation refers to the diagnostic criteria of the fourth version of the DSM, it is still used in research because in the updated manual (DSM-5) the symptoms and cutoff score for diagnosing this disorder remain unchanged. Hence, to state that the child's score indicates inattention or hyperactivity/impulsivity, the parent/guardian needs to have marked six or more symptoms in each category as “very much” or “too much”. In this study, children were considered to present scores indicating inattention and/or impulsivity/hyperactivity, or not to present this, in accordance with the responses given by their parent/guardian.

### Statistical analysis of the data

The data analysis employed the following statistical procedures. Descriptive statistics were used to characterize the results. In making statistical inferences, a machine-learning technique (logistic regression with LASSO) was used to evaluate which variables were risk or protective factors for the development of language and/or arithmetic skills. The significance level used for the logistic regression models was α = 0.05.

A logistic regression is defined as where is the probability of a certain class or event such as pass/fail, alive/dead or healthy/sick existing for the *i*-th individual, is the value of the *j*-th variable for the *i*-th individual and s are the regression coefficients. The variables can be quantitative (continuous or discrete) or binary, taking values of 1 if the *i*-th individual has a specific feature or 0 otherwise. A categorical variable with *d* classes is usually represented by *d-1* binary variables in this model. The regression coefficients are estimated based on the observed sample through maximum likelihood or least-squares methods.

In general, only the variables that are significant (risk factors) are kept in the model to explain the probability. In this study, we selected the significant variables using LASSO, a machine-learning method, estimated their regression coefficients using the maximum likelihood method and verified whether they were really significant at the significance level 0.05. The LASSO method consists of estimating the regression coefficients through the least-squares method, including the restriction, where *t* is a prespecified positive value. This restriction forces the regression coefficient of non-significant variables to be zero, and they can then be removed from the model.

## RESULTS

Among the 66 participating children, 17 (26%) were classified as “average” for language and arithmetic skills, whereas 28 (42%) had alterations in both. The other 21 children (32%) had an alteration in either language or arithmetic. **Table 2** provides a description (age, sex and school year) of the children who formed groups 1 and 2 in each statistical inference model (model for language ability and model for arithmetic skill).

**Table 2:** Description (age, sex and school year) of the children who formed groups 1 and 2, according to performance in language and arithmetic

	Language (oral and written language)		Arithmetic skills	
	Group 1 (n = 41)	Group 2 (n = 25)	Group 1 (n = 36)	Group 2 (n = 30)
Female	20	7	16	14
Male	21	18	20	16
6 years old	3	3	5	1
7 years old	6	3	6	3
8 years old	10	9	9	10
9 years old	15	8	11	12
10 years old	4	1	2	3
11 years old	3	1	3	1
First year	6	2	7	1
Second year	7	6	9	4
Third year	16	7	12	11
Fourth year	7	6	4	9
Fifth year	5	4	4	5

The two logistic regression models are presented below. The first (**Table 3**) shows the results regarding language skills: group 1 = 41 children (62%) with language alterations and group 2 = 25 (38%) without language alterations. The second (**Table 4**) shows the results regarding arithmetic skills: group 1 = 36 children (55%) with arithmetic alterations and group 2 = 30 (45%) without arithmetic alterations.

**Table 3:** Logistic regression model with LASSO, analyzing which variables were risk or protective factors for linguistic development (oral and written language)

	Estimatedcoefficient	P-value
Adequate arithmetic performance	2.7	0.004*
Gender - female	-1.7	0.02*
Hyperactivity/impulsivity	-1.6	0.07
Mother's schooling level - higher education	1.9	0.04*
Age when pregnant < 18	-2.1	0.02*
Consumption of psychoactive substances during pregnancy	-3.1	0.01*
History of otitis in early childhood	-2.3	0.02*

Logistic regression test with LASSO (α = 0.5)

* Statistically significant variables. A positive coefficient is a protective factor, i.e. the presence of this variable increases the possibility of adequate linguistic development. A negative coefficient is a risk factor, i.e. the presence of this variable is a risk for language alterations. Note: The test results are presented only as the P-value and the estimated coefficient of the significant variables with α = 0.1. Variables selected for this study: mother's age during pregnancy (≤ 18 years = teenage pregnancy); consumption of psychoactive substances during pregnancy (alcoholic beverages, tobacco and other drugs); complications during pregnancy (e.g. physical trauma, surgery, bleeding and others); mother's schooling level; home environment resources; gestational age (preterm, full-term or post-term); size for gestational age (weight and length); birth complications; delay in neuropsychomotor development; delay in speech and language development; epilepsy and other conditions in early childhood (malnutrition, anemia, etc.); current complaints of sleep alterations; history of recurrent otitis or serous otitis in early childhood; alterations in social skills; and alterations in behavior.

**Table 4:** Logistic regression model with LASSO, analyzing which variables are risk or protective factors for arithmetic skills

	Estimated coefficient	P-value
Adequate language performance	2.4	0.01*
Alteration in responsibility skill	-7.5	0.01*
Alteration in civility skill	-3.7	0.01*
Inattention	-3.1	0.03*
Mother's schooling level - higher education	4.5	0.02*
Age when pregnant < 18	-2.6	0.03*
Complications during pregnancy	-2.6	0.05*
Absence of delay in speech and language development	3.3	0.03*

Logistic regression test with LASSO (α = 0.5)

* Statistically significant variables. A positive coefficient is a protective factor, i.e. the presence of this variable increases the possibility of adequate mathematical performance. A negative coefficient is a risk factor, i.e. the presence of this variable is a risk for alterations in arithmetical skills. Note: The test results are presented only as the P-value and the estimated coefficient of the significant variables with α = 0.1. Variables selected for this study: mother's age during pregnancy (≤ 18 years = teenage pregnancy); consumption of psychoactive substances during pregnancy (alcoholic beverages, tobacco and other drugs); complications during pregnancy (e.g. physical trauma, surgery, bleeding and others); mother's schooling level; home environment resources; gestational age (preterm, full-term or post-term); size for gestational age (weight and length); birth complications; delay in neuropsychomotor development; delay in speech and language development; epilepsy and other conditions in early childhood (malnutrition, anemia, etc.); current complaints of sleep alterations; history of recurrent otitis or serous otitis in early childhood; alterations in social skills; and alterations in behavior.

The results from the statistical model investigating the relationship between the development of language (oral and written) and several variables (**Table 3**) showed that the mother's age when pregnant, mother's schooling level and events during pregnancy were important variables. The mothers of children with good language development had completed higher education. However, young maternal age at pregnancy (teenage pregnancy), gestational complications and consumption of psychoactive substances during pregnancy are variables that compromise adequate language development. Being female and having a history of ear infections were also variables negatively related to language development.

Adequate development of arithmetic skills was positively influenced (protective factors) by the variables of higher mother's schooling (higher education) and adequate linguistic development, along with not having any history of delay in the child's first words. Young maternal age during pregnancy and complications/substance use during pregnancy were also risk factors (negative association) for development of arithmetic skills, but in addition, we found that the variables of altered social skills (civility or responsibility) and symptoms of inattention were risk factors. These variables were not significant in the model for linguistic ability (**Table 4**).

## DISCUSSION

The results from this study indicated what the risk and protective factors for the development of language and arithmetic skills are. It was observed that adolescent pregnancy (mother 18 years old or younger during pregnancy), consumption of psychoactive substances and complications during pregnancy were risk factors for the development of both skills studied here. The mother's schooling level (higher education), on the other hand, was a protective factor.

Regarding language development alone, being female and having a positive history of recurrent otitis in early childhood were risk factors. However, having adequate performance in arithmetic was a protective factor.

Arithmetic skill, in turn, was negatively influenced by complaints of inattention, and alterations in the social skills of responsibility and civility. In contrast, adequate language development (no history of speech delay and average current linguistic performance) was a protective factor.

The first datum to be discussed is the prevalence of low school performance. In this study, only 26% of the children had adequate linguistic and arithmetic performance. The others had some degree of impairment, either mildly (dysorthography) or severely (illiterate children). These data are similar to those from other studies conducted in Brazil and are also in agreement with data published by federal government agencies, through the Primary Education Assessment System (SAEB).^5^^,^^17^ According to data from the SAEB, the number of children with adequate reading and arithmetic performance by the end of fifth grade is lower than 20%.

Regarding protective factors, there is a positive relationship between language and arithmetic development. Analysis has been conducted on cognitive differences between children with alterations only in reading/writing, those with alterations only in arithmetic and those with alterations in both skills.^18^ It was observed that children with more complex cognitive alterations (as in working memory, phonological awareness, numerical sense and others) are the ones that have difficulties in both domains (language and arithmetic). However, children with difficulties only in arithmetic also presented some alterations in linguistic skills, such as narrative memory. Other studies have shown that to develop arithmetic skills it is necessary to develop certain linguistic skills.^2^^,^^19^

Regarding environmental variables (mother's schooling, home environment resources and mother's age during pregnancy), this study showed that these can be risk or protective factors. Teenage pregnancy is considered to be a public health issue in Brazil, where it is estimated that one out of every five children is born of a teenage mother.^20^ In some regions of Brazil, this rate can reach 50%.^21^ In this study, this variable was considered to be a risk factor for language and arithmetic development.

Teenage pregnancy is also related to lower socioeconomic level and to difficulties that teenage mothers face in trying to study further and even to reach higher education, which is considered to be a protective factor.^20^^,^^21^ This is a high-risk scenario, in which teenage mothers are less likely to finish their education, thus resulting in low schooling levels.

Furthermore, there are home environment resources, which were not a significant variable in this study. However, it has been demonstrated in the literature that there is a positive relationship between environmental resources and linguistic/cognitive development.^13^^,^^22^ In an environment where adults have low schooling, it is common not to find many resources/opportunities for their children's development.^13^^,^^22^

Consumption of psychoactive substances such as alcoholic beverages, tobacco or other types of drugs while pregnant, and its negative relationship with development has been widely addressed in the literature. Review studies have concluded unanimously that consuming psychoactive substances during pregnancy causes functional and/or organic alterations in the central nervous system, which lead to alterations in the auditory pathway, and in motor and cognitive development.^23^^–^^25^

Complaints of inattention, which were considered in this study to be a risk factor for arithmetic skills, may be signs of alterations in the central nervous system. These could be caused through consumption of substances during pregnancy, genetic issues or other factors.^26^ Attentional complaints may point to a diagnosis of attention-deficit/hyperactivity disorder or mood alterations,^26^ which have high rates of comorbidities relating to learning difficulty.^5^^,^^6^ Whenever attentional complaints and/or externalizing behaviors are present, it is essential to make a differential diagnosis and institute adequate treatment.

Prematurity, which has been pointed out as a risk factor for linguistic and cognitive development, was not a significant variable in this study. One possible explanation for this is that prematurity would not be a risk factor in itself: instead, the risk would be due to other factors related to this.^9^ Premature children whose language development was at risk were found in a previous study to be those who had peri-intraventricular hemorrhage or bronchopulmonary dysplasia, birth weight lower than 1,000 grams and long hospitalization.^9^ On the other hand, low-risk premature children, i.e. those without a history of the abovementioned variables, usually have adequate language and writing development.^10^

Regarding gender, the literature indicates that primary language disorders (language development disorder, specific learning disorder, childhood apraxia of speech and others) are more prevalent in males.^26^ However, in the present study, being female was a risk factor for language development.

One possible explanation for this finding is that the children sampled in this study had alterations mostly in written language. However, the purpose of the present study was not to verify the differential diagnoses of the causes underlying language alteration, but to ascertain the risks in a sample with language alterations, regardless of their degree and cause. Therefore, and also based on the literature regarding the percentage of children with primary language alterations,^5^^,^^6^^,^^26^ it can be suggested that the risk factor for language that relates to being female depends on other factors not investigated here, such as mood alterations, which females more often present, along with other factors.^26^

A history of otitis in early childhood is related to difficulties in phonological development,^27^ which in turn is related to the metalinguistic skill of phonological awareness. This essential skill for written language development was assessed in the present paper.^1^^,^^18^

Lastly, there are social skills. In this study, difficulties regarding responsibility and civility skills were observed in relation to arithmetic alterations. Alterations in social skills may be present in 20% to 42% of the children with school difficulties.^28^ Deficits in the skills needed to make and maintain friends, end a conversation, play together with other children and interact with classmates are characteristic of children with learning difficulties. Moreover, these deficits tend to affect friendships, as well as important behaviors related to school learning, e.g. asking questions, having questions answered and asking a classmate or teacher's help.^28^^,^^29^

A piece of research demonstrated that the ability to write to dictation is positively correlated with civility and altruism skills; assertiveness, in turn, was correlated with arithmetic skills.^29^ An intervention study demonstrated that children who had received an intervention to train their social skills improved in this area and in academic skills as well. In contrast, children who had received reading and writing interventions only improved in that educational area.^30^

## CONCLUSION

Only 26% of the children enrolled in this elementary school (first to fifth grades) had adequate language and arithmetic development. The others had some type of alteration that ranged from mild to severe deficits. These data confirm the sad reality of Brazilian public education.

Among the variables analyzed, i.e. social skills and maternal/environmental variables (mother's age during pregnancy, mother's schooling, environmental resources and others; behavioral problems/inattention and hyperactivity complaints; pre, peri and postnatal complications; and neuropsychomotor development), the risk factors for both skills (language and arithmetic) were those relating to gestation (consumption of psychoactive substances or complications during that period) and teenage pregnancy. The protective factor, on the other hand, was the mother's schooling level (higher education).

For the linguistic model, being female and having a history of otitis were also risk factors. For arithmetic, alterations in the social skills of responsibility and civility, as well as inattention complaints, were risk factors. The protective factor in the arithmetic model was adequate linguistic development.

Regarding impairment of social skills and inattentive behavior, it was not possible to establish a clear link of cause and effect, since we did not investigate whether these changes preceded the arithmetic trouble or vice versa. Hence, we can only conclude that they are related. Thus, children with difficulties in arithmetic have more attentional complaints and deficiencies in specific social skills.
